# Integrative Analysis of 4-Hydroxynonenal-Modified Proteins and Plasma Metabolome in Breast Cancer Patients

**DOI:** 10.3390/antiox15020265

**Published:** 2026-02-21

**Authors:** Morana Jaganjac, Matea Nikolac Perkovic, Tea Horvat, David Rojo, Marija Krizic, Natalija Dedic Plavetic, Damir Vrbanec, Biserka Orehovec, Kamelija Zarkovic, Neven Zarkovic

**Affiliations:** 1 Division of Molecular Medicine, Ruder Boskovic Institute, 10000 Zagreb, Croatia; matea.nikolac.perkovic@irb.hr (M.N.P.); tea.horvat@irb.hr (T.H.); 2 Centro de Metabolómica y Bioanálisis (CEMBIO), Facultad de Farmacia, Universidad CEU San Pablo, Campus Montepríncipe, ES-28003 Madrid, Spain; david.rojoblanco@ceu.es; 3 University Hospital Centre Zagreb, Kispaticeva 12, 10000 Zagreb, Croatia; mkrizic13@gmail.com (M.K.); ndedic@kbc-zagreb.hr (N.D.P.); kamelijazarkovic@gmail.com (K.Z.); 4 School of Medicine, University of Zagreb, Kispaticeva 12, 10000 Zagreb, Croatia; 5 Juraj Dobrila University of Pula, 52100 Pula, Croatia; dvrbanec197@gmail.com; 6 Clinical Hospital Dubrava, 10000 Zagreb, Croatia; biserka.orehovec@gmail.com

**Keywords:** breast cancer, lipid peroxidation, 4-hydroynonenal, metabolome remodeling

## Abstract

Breast cancer is a highly heterogeneous malignancy, characterized by diverse genetic, epigenetic, and phenotypic variations, as well as by metabolic reprogramming and oxidative stress. Lipid peroxidation bioactive product 4-hydroxynonenal (4-HNE) plays a significant role in the development and progression of cancer. In this study, we quantified circulating 4-HNE-modified proteins and performed comprehensive untargeted metabolomic profiling of the patients’ plasma using LC-ESI-QTOF-MS and GC-EI-QMS, aiming to investigate systemic metabolic pathways associated with oxidative damage in breast cancer. Significantly elevated levels of 4-HNE-modified proteins were detected in breast cancer patients compared to healthy controls, accompanied by distinct metabolomic signatures enriched in lipid metabolism. Several metabolites, including specific long-chain fatty acids, exhibited significant correlations with circulating 4-HNE-modified proteins, suggesting an interaction between lipid peroxidation-driven protein modification and breast cancer-associated metabolic reprogramming. Overall, this study provides evidence of associations between systemic 4-HNE-mediated protein modification and altered metabolic profiles in breast cancer, highlighting oxidative stress–related metabolites as potential biomarkers and pointing to redox-metabolic crosstalk in breast cancer patients.

## 1. Introduction

Breast cancer remains the most frequently diagnosed malignancy and a leading cause of cancer-related mortality among women worldwide, representing a major public health burden and a persistent clinical challenge. Given the heterogeneity of breast cancer and the limitations of current diagnostic methods, identifying reliable and minimally invasive biomarkers that capture its underlying biochemical complexity is essential for improving early detection, patient stratification, and treatment monitoring. In recent years, metabolomics has emerged as a powerful analytical approach for characterizing the small-molecule signatures associated with tumor development and progression. Cancer cells undergo profound metabolic reprogramming to support proliferation, survival, and metastasis. As a result, the plasma metabolome can serve as a reliable reflection of the dynamic interface between tumor activity and systemic physiology. Numerous studies have shown that breast cancer significantly alters circulating metabolites, highlighting the potential of metabolomics to identify clinically relevant biomarkers. For example, aberrant amino-acid metabolism, particularly involving glutamine, glutamate, alanine, aspartate, and branched-chain amino acids, has been consistently observed across multiple cohorts. These changes likely reflect enhanced glutaminolysis and nitrogen turnover that support tumorigenesis [1,2]. Alterations in other metabolic classes, including carbohydrates, carboxylic acids, and lipids such as phosphatidylcholines, sphingomyelins, triacylglycerols, and acylcarnitines, further underscore the diverse metabolic adaptations characteristic of breast cancer [3,4,5,6]. These metabolic signatures not only reflect tumor-specific biochemical demands but also offer potential diagnostic and prognostic value. As metabolism is tightly linked to cellular redox balance, oxidative stress represents a central component of cancer-associated metabolic dysregulation. Reactive oxygen species (ROS) are elevated in breast cancer due to mitochondrial dysfunction, altered metabolic flux, and an inflammatory microenvironment. One of the most biologically active and extensively studied lipid peroxidation products generated under oxidative stress is 4-hydroxynonenal (4-HNE). This electrophilic aldehyde can modify proteins, nucleic acids, and lipids, thereby influencing diverse cellular processes including proliferation, apoptosis, angiogenesis, immune modulation and metabolic rewiring [7,8]. In breast cancer, 4-HNE has been implicated in stabilizing HIF-1α, promoting VEGF-mediated angiogenesis, suppressing tumor-suppressor pathways such as PTEN, and modulating mitochondrial enzymes that regulate redox homeostasis [9,10]. Elevated levels of 4-HNE-protein adducts have been reported in the plasma and tissues of breast cancer patients, suggesting its potential use as an indicator of malignant transformation [11,12].

Although plasma metabolomics has revealed metabolic alterations in breast cancer and independent studies have documented altered 4-HNE levels in affected patients, no studies to date have systematically examined the relationship between 4-HNE and circulating metabolic alterations in breast cancer. This represents a critical knowledge gap because 4-HNE not only reflects oxidative damage but it may also functionally modify metabolic enzymes and regulatory proteins, suggesting that its systemic abundance may influence, or be influenced by, metabolic changes associated with tumor progression. Understanding this interplay could yield new mechanistic insights into the biology of breast cancer. Therefore, we conducted a comprehensive analysis that integrated plasma metabolomics with measurements of 4-HNE-modified proteins in breast cancer patients to define metabolite-4-HNE associations and identify metabolic alterations in breast cancer.

## 2. Materials and Methods

### 2.1. Chemicals and Reagents

Chemicals and reagents used for metabolomic analyses were all LC-MS grade and included acetonitrile, formic acid, heptane, O-methoxyamine hydrochloride, pyridine, tricosan, and 4-chlorophenol (used as internal standards in GC-MS analysis). All solvents and reagents were obtained from Sigma-Aldrich (St. Louis, MO, USA), except for N,O-bis(trimethylsilyl)trifluoroacetamide (BSTFA) containing 1% trimethylchlorosilane (TMCS; Pierce Chemical Co., Rockford, IL, USA), the fatty acid methyl ester (FAME) standard mixture (Supelco, Bellefonte, PA, USA), and the API-TOF reference mass solution kit (Agilent Technologies, Waldbronn, Germany). Ultrapure water was obtained from the MilliQ^®^plus185 system (Millipore, Billerica, MA, USA).

### 2.2. Study Participants and Sample Collection

This study was conducted in accordance with the approval of the Ethics Committee of the University Hospital Centre Zagreb (approval code 02/21AG), and all participants provided written informed consent. This prospective cohort study included 41 female patients with breast cancer diagnosed at the University Hospital Centre Zagreb in the period from 23 August 2017, to 13 February 2018, as well as 31 healthy female controls matched for age and other relevant baseline characteristics. Before inclusion in the study, all patients had a histopathologically confirmed diagnosis and had not initiated any treatment before blood collection for metabolomic analysis. Peripheral blood samples were collected via venipuncture into EDTA tubes containing BHT. Samples were centrifuged at 3000× *g* for 20 min at 4 °C to obtain plasma, which was stored at −80 °C until further analysis. Control plasma was obtained from age-matched healthy volunteers following the same protocol.

### 2.3. Plasma 4-HNE ELISA

Plasma levels of 4-HNE-protein adducts were quantified using a previously established in-house ELISA protocol [13]. Briefly, 10 μL of plasma (adjusted to 10 mg/mL protein) or standards were added to 100 μL of carbonate buffer (0.05 M, pH 9.6) in triplicate wells of Nunc Immuno Maxisorp plates and incubated for 5 h at 4 °C. Wells were blocked with 5% fat-free dry milk in carbonate buffer for 3 h at room temperature before overnight incubation with the same monoclonal anti-4-HNE antibody at 4 °C. After endogenous peroxidase quenching, wells were incubated with secondary antibody for 1 h at room temperature, followed by substrate addition and absorbance measurement at 450/620 nm. All steps included five washes with the wash buffer. Concentrations of 4-HNE-protein adducts were calculated against a standard curve and expressed as pmol 4-HNE/mg protein.

### 2.4. Sample Preparation and Metabolite Extraction

Plasma samples were processed and metabolites extracted following the procedure previously described in [14]. Briefly, for both GC-MS and LC-MS analysis, the plasma samples were deproteinized with acetonitrile. After deproteinization, the supernatant was transferred to crimp-top clear glass vials with inserts and evaporated to dryness. Methoximation was performed by adding O-methoxyamine hydrochloride, and the next day, BSTFA with 1% TMCS was added to each vial for silylation. Before the analysis, tricosane was added as an internal standard. In the case of LC-MS sample preparation, after deproteinization, the remaining supernatant was transferred to the crimp-top clear glass vials with inserts and analyzed. Individual quality control (QC) samples for both analyses were prepared by pooling and mixing equal volumes of each plasma sample. Both quality control and blank samples were prepared in the same way as plasma samples. Before metabolomic analysis, samples were randomized to minimize systematic bias. The injection sequence included blank samples at the beginning and the end, while QC samples were injected at the start to condition the column. During the analysis, after every 10 samples, one QC sample was injected to monitor instrument performance. The entire sequence was run continuously in a single batch.

### 2.5. GC-MS Analysis and Data Processing

The Agilent 7890A gas chromatograph equipped with an autosampler (Agilent Technologies 7693, Waldbronn, Germany) and coupled to an inert MSD with a quadrupole analyzer (Agilent Technologies 5975, Waldbronn, Germany) was used for GC-MS analysis. The method has been described in detail previously [14]. In brief, a 2 μL injection volume was applied with a split ratio of 1:10. A DB-5MS GC column with a pre-column was used for compound separation, with helium serving as the carrier gas. Each sample was analyzed for a total of 37.5 min, during which mass spectra were recorded across a mass range of 50–600 *m*/*z*. Agilent MassHunter Qualitative Analysis 10.0 software was used to assess the reproducibility of internal standard signals and the quality of the total ion chromatograms. Compound deconvolution and identification were performed using Agilent MassHunter Unknowns Analysis 10.0. Initial compound identification was based on retention time (RT) and mass spectra using the Fiehn library, followed by re-evaluation of both identified compounds and unknown features using the NIST library (National Institute of Standards and Technology, 2017 version). Peak integration was conducted with Agilent MassHunter Quantitative Analysis 10.0. For quantification of each compound, the area of a specific target ion (quantifier) was integrated, and this abundance (area under the peak, AUP) was normalized to the abundance of tricosane (internal standard) by dividing the AUP of each compound by the AUP of tricosane. The relative quantification relies on the principle that the AUP in a chromatogram is directly proportional to the amount of analyte injected. Before statistical analysis, the blank subtraction was also carried out.

### 2.6. LC-MS Analysis and Data Processing

The samples were also analyzed using an Agilent Technologies 1200 Series liquid chromatography system coupled to an Agilent 6520 Accurate-Mass Q-TOF detector (Agilent Technologies, Waldbronn, Germany). The analytical procedure has been previously described in detail [14]. In summary, metabolite separation was performed on a reversed-phase column (Discovery^®^ HS C18, 15 cm × 2.1 mm, 3 µm; Supelco, Bellefonte, PA, USA) equipped with a matching pre-column (Discovery^®^ HS C18, 2 cm × 2.1 mm, 3 µm; Supelco, Bellefonte, PA, USA). The injection volume was 10 µL, and the flow rate was set to 0.6 mL/min, applying a gradient using solvent A (water with 0.1% FA) and solvent B (ACN with 0.1% FA). All samples were analyzed in both positive and negative ESI modes. As with the GC-MS data, analytical quality was evaluated using the Agilent MassHunter Qualitative Analysis tool 10.0. The raw LC-MS data were then processed in Agilent MassHunter Profinder 10.0 for feature deconvolution using the Molecular Feature Extraction (MFE) algorithm followed by Recursive Feature Extraction (RFE). The resulting list of statistically significant accurate masses was annotated with the CEU Mass Mediator search tool [15], as previously described [14]. Tandem mass spectrometry (MS/MS) was conducted to support the identification of significant metabolites, using the same LC-MS setup and chromatographic conditions as the primary analysis. Selected ions were fragmented by multiple collision energies (10, 20, and 40 eV). Compound identities were confirmed by comparing the resulting fragmentation spectra with those in public MS/MS spectral libraries (HMDB, METLIN, LipidMaps). Identification criteria included consistent retention time, accurate mass (within ±20 ppm), and the presence of at least two characteristic MS/MS fragments (putatively annotated compounds, Metabolomics Standards Initiative level 2 identification).

### 2.7. Statistical Analysis

Before statistical analysis, the GC-MS and LC-MS raw data were filtered according to the criteria described by Godzien et al. [16]. Variables were retained if they appeared in ≥80% of the Quality Control (QC) samples with a relative standard deviation (RSD) < 30%, or if they appeared in < 20% of QC samples but were present in ≥50% of samples within a specific subject group. To correct potential intra-batch effects, we applied the Quality Control–Robust Spline Correction (QC-RSC) algorithm, as recommended by Kuligowski et al. [17]. Support vector regression for QC-RSC was performed using MATLAB (7.10.0.499, MathWorks, Natick, MA, USA) and the LIBSVM library [18]. All multivariate statistical analyses were carried out using SIMCA-P+ (version 15.0.2.5959; Umetrics, Umeå, Sweden). These analyses included Principal Component Analysis (PCA), Partial Least Squares–Discriminant Analysis (PLS-DA), and Orthogonal PLS-DA (OPLS-DA). Volcano plots were constructed from OPLS-DA results by plotting variable importance in projection (VIP) against corrected *p*-values [p(corr), loading values scaled as correlation coefficients]. Permutation analyses were performed for the obtained multivariate OPLS-DA models to assess their reliability. Possible overfitting of the OPLS-DA model was further evaluated by cross-validated ANOVA (CV ANOVA).

Univariate statistical analyses were performed in MATLAB (7.10.0.499). Normality was tested using the Kolmogorov–Smirnov test. Group comparisons were conducted using either Student’s *t*-test or Mann–Whitney U test, depending on data distribution, followed by Benjamini–Hochberg procedure in order to control the false discovery rate (FDR). The *p*-value correction was done across all detected features per platform/mode.

Differences in the levels of 4-HNE protein adducts between healthy controls and breast cancer patients were evaluated. Since data were not normally distributed (Shapiro–Wilk test, *p* < 0.05), the group differences were assessed using the Mann–Whitney U test. Associations between significantly altered metabolites and plasma levels of 4-HNE–modified proteins in both patient and control groups were assessed by Spearman’s rank correlation analysis. The correlations were computed within each group. All statistical analyses were two-tailed, and a *p*-value < 0.05 was considered statistically significant. Additionally, we have applied a false discovery rate (FDR) correction (Benjamini–Hochberg method) to all correlation *p*-values.

Metabolites were considered significantly altered if they met the criteria *p* ≤ 0.050 (Benjamini–Hochberg adjusted), VIP ≥ 1.00, and |p(corr)| ≥ 0.30. Fold change was calculated as the ratio of the mean metabolite abundance in the breast cancer cohort to that of the healthy control group. Percentage change (%Δ) was calculated as: [(mean CASE − mean CONTROL)/mean CONTROL] × 100, with positive values indicating increased abundance and negative values indicating decreased abundance in the CASE (breast cancer) group relative to the CONTROL group.

## 3. Results

**Figure 1:**
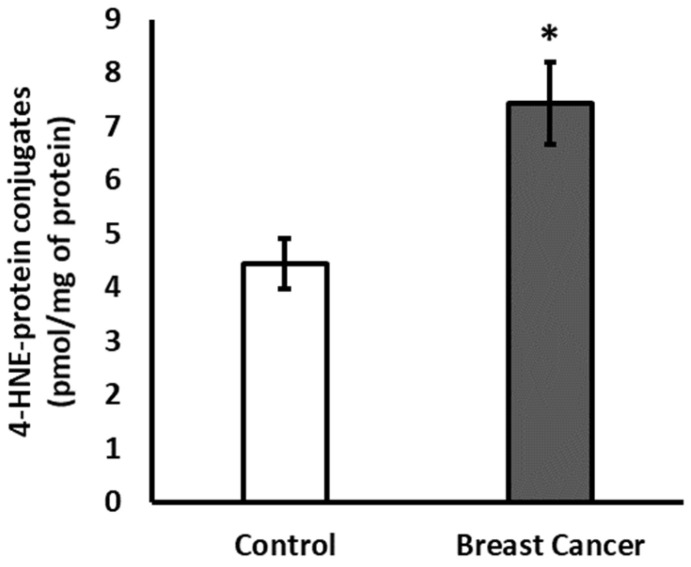
The amount of plasma proteins modified by 4-HNE. Data are presented as mean ± SEM. Significance: * *p* < 0.05.

This study included 41 patients with histopathologically confirmed breast cancer and 31 healthy controls to investigate the impact of 4-HNE on circulating proteins and the plasma metabolome. The median age of the breast patients was 57 years (IQR 50–67.5), and for the healthy controls, 56 years (IQR 52–62.5). Most breast cancer patients were postmenopausal (68%) and overweight or obese (49% overweight and 12% obese) with invasive breast cancer (95.1%). The majority of patients had early-stage HR-positive, HER2-negative breast cancer (48.7%), followed by HER2-positive (28.2%) and triple-negative cancers (17.9%). Baseline demographic and clinical characteristics of breast cancer patients are presented in Supplementary Table S1. A marked elevation of 4-HNE-modified plasma proteins was observed in breast cancer patients (mean 7.4 [IQR: 4.6–9.4] pmol/mg protein, 95% CI: 5.9–9.0) compared with healthy controls (mean 4.4 [IQR: 2.3–6.6] pmol/mg protein, 95% CI: 3.4–5.4) (Figure 1). Group differences assessed by the Mann–Whitney U test confirmed a statistically significant increase in breast cancer patients (*p* = 0.006), indicating significantly higher systemic lipid peroxidation–associated protein modification.

**Figure 2:**
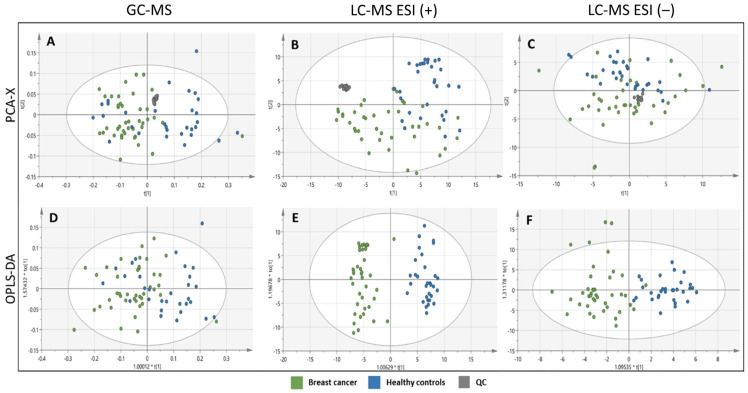
Differences in plasma metabolite profiles between the breast cancer group (green) and healthy controls (blue). PCA and OPLS-DA score plots were obtained using SIMCA-P+ software (version 15.0.2.5959, Umetrics, Umea, Sweden). (**A**) PCA score plot for the GC-MS analysis (R2X(cum) = 0.984; Q2(cum) = 0.465); (**B**) PCA score plot for the LC-MS ESI (+) analysis (R2X(cum) = 0.268; Q2(cum) = 0.204); (**C**) PCA score plot for the LC-MS ESI (−) analysis (R2X(cum) = 0.211; Q2(cum) = 0.126); (**D**) OPLS-DA score plot for the GC-MS analysis (R2X(cum) = 0.318; R2Y(cum) = 0.670; Q2(cum) = 0.449; p(CV-ANOVA) = 6.46 × 10^−7^); (**E**) OPLS-DA score plot for the LC-MS ESI (+) analysis (R2(cum) = 0.418; R2Y(cum) = 0.903; Q2(cum) = 0.644; p(CV-ANOVA) = 1.68 × 10^−10^); (**F**) OPLS-DA score plot for the LC-MS ESI (−) analysis (R2X(cum) = 0.325; R2Y(cum) = 0.800; Q2(cum) = 0.439; p(CV-ANOVA) = 3.00 × 10^−6^).

Profiling of the plasma metabolome revealed distinct metabolic signatures differentiating breast cancer patients from healthy controls (Figure 2). PCA was performed to assess the unsupervised separation between healthy controls and breast cancer patients (Figure 2). The tight clustering of QC samples across all analyses confirms the robustness of the analytical workflow and demonstrates that data normalization effectively corrected any instrumental variability, supporting that the observed differences between clinical groups are biological in nature (Figure 2). Following PCA, supervised OPLS-DA models were constructed to distinguish the two sample groups, and variables with the highest discriminative power were identified through VIP scoring (Table 1). The OPLS-DA models clearly demonstrated a separation between breast cancer and control subjects (Figure 2).

PCA score plots (Figure 2) suggest modest clustering between the two subject groups, especially in the case of LC-MS results. The OPLS-DA score plots (Figure 2) revealed moderate clustering between the two subject groups, as reflected by Q2 values below 0.5 for both GC-MS and LC-MS ESI (−) analyses. In contrast, for the LC-MS ESI (+) analysis, the Q2 value, which reflects the model’s predictive capability, indicates good predictive performance. All OPLS-DA models were built from one predictive component and two orthogonal components (Figure 2). Permutation analysis (500 iterations) was performed to validate all OPLS-DA models by randomly shuffling class labels to assess whether the observed predictive ability (Q^2^) and goodness-of-fit (R^2^) were genuine or occurred by chance (Figure S1). The permutation analyses indicated that the original models are valid since the criteria for validity were satisfied (all permuted Q2-values to the left are lower than the original points to the right, and the blue regression line of the Q2-points intersects the vertical axis below zero) (Figure S1). As supplementary material, we also included scatter plots of the cross-validated (CV) score vectors that are analogous to the scatter plot of regular score vectors (Figure S2). These plots indicate how sensitive each model is to the exclusion of an observation from the work set.

Using GC-MS analysis, a total of 83 signals were detected, while the LC-MS analysis resulted in 1286 features detected in the positive mode and 1324 in the negative ionization mode. After normalization of the raw data matrix, curation of the data, statistical analysis, and metabolite identification, a total of 36 metabolites detected with GC-MS and/or LC-MS were significantly altered between healthy control subjects and breast cancer patients (Table 1). Only metabolites that had VIP ≥ 1.00 and pBH < 0.050 were taken into consideration (Table 1). After GC-MS-based metabolomic profiling, we detected only 7 metabolites that were significantly altered in patients with breast cancer compared to healthy controls (Table 1). These metabolites included two fatty acyls (caproic and stearic acid), lactic acid, 2-hydroxybutyric acid, pyruvic acid, mannose, and cholesterol. The LC-MS-based metabolite profiling of plasma samples from breast cancer patients detected numerous features. A total of 30 metabolites were found by LC-MS/MS analysis to be significantly altered in breast cancer patients compared to healthy controls, with the majority belonging to lipids, particularly fatty acyls (Table 1). Stearic acid emerged as a significantly altered compound in both GC-MS and LC-MS analyses, with the same pattern of change (Table 1).

Metabolites found to be significantly altered in breast cancer patients compared to healthy controls primarily included fatty acyls, glycerophospholipids, sterol lipids, organoheterocyclic compounds, and organic acids and their derivatives (Table 1). The results indicate an increase in different fatty acyls in patients with breast cancer compared to healthy subjects, except for caproic acid. Several long-chain fatty acids (LCFAs) were found to be significantly altered compared to healthy controls. Among the non-oxidized LCFAs, stearic acid (octadecanoic acid), methylheptadecatrienoic acid, eicosapentaenoic acid, docosapentaenoic acid, anandamide (18:3), and undecanoylcarnitine were identified, with docosapentaenoic acid exhibiting the highest fold change, more than tenfold compared to controls. Stearic acid was detected by both GC-MS and LC-MS, and in both analytical approaches, it was found to be more abundant in patients with breast cancer than in healthy controls, showing comparable fold changes (Table 1). In parallel, several oxidized or derivatized LCFAs were significantly elevated, including 14-HDoHE, epoxyeicosatrienoic acids, octadecadienal, and epoxyoctadecenoic acid, with 14-HDoHE and epoxyeicosatrienoic acids showing the most pronounced increases, also exceeding a tenfold change. Additionally, certain monoacylglycerols and oxidized cytidine-diphosphate-diglycerides (CDP-DG) were more abundant in breast cancer patients. In addition, two metabolites appeared to be specific to the breast cancer cohort. Unidentified metabolites are shown in Supplementary Table S2.

To investigate whether the elevated abundance of 4-HNE–modified plasma proteins may contribute to broader metabolic disturbances, we assessed correlations between 4-HNE adduct levels and metabolites that showed significant alterations in breast cancer. This correlation analysis, performed across samples from both patients and healthy controls, is presented in Table 2.

In breast cancer patients, levels of plasma 4-HNE protein conjugates showed strong positive correlations with several metabolites associated with lipid peroxidation and steroid metabolism, including 14-HDoHE, androstanediol, pregnenolone, and epoxyeicosatrienoic acids, indicating a potentially clinically relevant correlation. Moderate positive associations were also observed between 4-HNE and 4,5-dehydro-docosahexaenoic acid, docosapentaenoic acid, dynorphin B (6–9), eicosapentaenoic acid, epoxyoctadecenoic acid, oxidized CDP-DG, PC(18:1), and retinal, suggesting possible coordinated alterations in oxidized lipid species and lipid-derived signaling molecules. Additionally, weaker but still positive correlations were detected for MG(18:0) and MG(18:2(9,12)).

In contrast, among healthy controls, 4-HNE displayed a different metabolic association pattern, showing moderate negative correlations with caproic acid and lactic acid, and a strong negative correlation with tetradecenoylcarnitine. However, after FDR adjustment, none of the correlations in healthy controls remained significant (pBH > 0.05). Interestingly, although MG(18:0) and tetradecenoylcarnitine did not show significant differences between breast cancer and control groups, 4-HNE levels exhibited notable correlations with these metabolites in breast cancer samples.

## 4. Discussion

Elevated levels of 4-HNE-modified plasma proteins in breast cancer patients indicate increased systemic oxidative stress and lipid peroxidation. A similar increase has been reported in other malignancies, including prostate cancer, lung cancer, brain tumors, colorectal cancer and ovarian cancer [7,14,19,20]. Metabolomic profiling revealed that breast cancer patients have markedly reduced caproic acid, whereas stearic acid showed a clear increase. This metabolic pattern mirrors our recent observations in patients with prostate cancer [14]. Several other metabolites exhibited similar cross-cancer behavior. Lactic acid and pyruvic acid (2-oxopropanoic acid) were consistently decreased, while 2-hydroxybutyric acid, mannose, and cholesterol were elevated in patients with malignancy compared with healthy controls. Moreover, a group of lipid-related metabolites, including eicosapentaenoic acid, docosapentaenoic acid, octadecadienal, MG(18:2(9,12)), biliverdin, and retinal, also showed increased levels in both breast and prostate cancer [14]. These alterations across different tumor types suggest shared metabolic reprogramming that may be characteristic of malignant transformation. Furthermore, the obtained results indicate that both the pool of non-oxidized fatty acids and their oxidized derivatives are markedly perturbed in breast cancer, highlighting extensive lipid metabolic remodeling and oxidative stress.

While elevated circulating 4-HNE-protein levels have been reported in multiple malignancies, our study highlights distinctive metabolic correlations in breast cancer that have not been previously described. In breast cancer patients, 4-HNE showed strong positive correlations with 14-HDoHE, androstanediol, pregnenolone, and epoxyeicosatrienoic acids. Moderate positive correlations were observed with docosapentaenoic acid, dynorphin B (6–9), eicosapentaenoic acid, epoxyoctadecenoic acid, oxidized CDP-DG, PC(18:1), and retinal. Weak positive correlations were observed for MG(18:2(9,12)), suggesting that 4-HNE levels reflect a complex metabolic milieu encompassing n-3 PUFA derivatives, steroid metabolism, and oxidized glycerophospholipids [21,22,23]. These associations were absent or markedly different in healthy controls, indicating disease-specific metabolic rewiring.

Positive associations with docosapentaenoic acid-derived metabolites (14-HDoHE, docosapentaenoic acid) and eicosanoids such as epoxyeicosatrienoic acids suggest that oxidative stress in breast cancer is linked to both n-3 and n-6 PUFA metabolic pathways. These alterations may influence tumor progression and the tumor microenvironment. Androstanediol, a metabolite of dihydrotestosterone, is known to promote proliferation of estrogen receptor-positive breast cancer cells in the absence of estradiol, indicating its potential role in breast cancer pathobiology [24]. Pregnenolone, a precursor to various steroid hormones, and retinal, a form of vitamin A, have not been directly linked to breast cancer. Nevertheless, their roles in hormone synthesis and cellular differentiation may indirectly influence cancer biology [24]. Correlations with androstanediol and pregnenolone further indicate an intersection between 4-HNE and dysregulated steroidogenesis, which is particularly relevant in hormone-responsive breast cancers.

Overall, our data suggest that breast cancer is associated with a systemic metabolic shift in which elevated 4-HNE is linked to increased oxidative metabolism of both n-3 and n-6 PUFAs, altered steroid pathways, and glycerophospholipid remodeling.

While the main limitation of this study is the relatively small number of patients and their heterogeneity, an additional important limitation is the correlative nature of the observed associations between circulating metabolites and 4-HNE levels. Nevertheless, this study has important clinical relevance as it highlights metabolites that are altered in patients with breast cancer. Eventually, these findings may also contribute to the identification of non-invasive biomarkers for breast cancer detection and an improved understanding of breast cancer initiation and progression. Finally, this is the first study showing an association between elevated 4-HNE levels and specific metabolomic alterations in the blood of patients with breast cancer, providing novel evidence for oxidative stress–related mechanisms of cancer growth and tumor–host relationships in these patients. Further mechanistic studies are warranted to determine whether these metabolic correlations are causal in tumor progression or reflective of broader oxidative stress-mediated remodeling.

## 5. Conclusions

In conclusion, our findings reveal consistent metabolic disturbances in breast cancer that partially parallel alterations previously observed in prostate cancer, suggesting shared features of metabolic reprogramming across different malignancies. Importantly, we identify distinct metabolic correlations between circulating 4-HNE and specific metabolites in breast cancer that have not been previously reported. Distinct correlation patterns between 4-HNE and numerous metabolites in breast cancer indicate disease-specific metabolic rewiring linking lipid peroxidation to PUFA oxidation, steroidogenesis, and glycerophospholipid turnover. Although several metabolites were not significantly altered between groups, their meaningful correlations with 4-HNE underscore their involvement in oxidative stress-associated metabolic networks. These results identify a set of metabolites that may contribute to a multi-analyte biomarker panel for cancer detection or classification. They also highlight potentially relevant clinical signatures specific to breast cancer, emphasizing the novel role of lipid peroxidation and steroid metabolism in this malignancy. While these findings are promising, validation in larger patient cohorts is required before clinical translation.

## Figures and Tables

**Table 1:** Significantly altered metabolites detected by GC-MS and/or LC-MS between patients with breast cancer and healthy control subjects. Significance was defined as pBH < 0.050 and VIP ≥ 1.00.

Category	Compound	Platform (Mode)	*m*/*z*	RT	FC	%Δ	log_2_FC	pBH
Carboxylic acids and derivatives	aconitic acid	LC-MS (−)	173.0100	0.79	1.27	27.13	0.35	0.040
Fatty Acyls	caproic acid	GC-MS	73.1	7.06	0.40	−60.14	−1.33	<0.001
	stearic acid	GCMS/ LC-MS (+)	341.3/ 285.2797	20.69/ 32.74	1.92/ 1.69	92.12/ 69.02	0.94/ 0.76	0.049/ 0.044
	methylheptadecatrienoic acid	LC-MS (+)	285.2797	29.42	2.53	153.14	1.34	0.007
	eicosapentaenoic acid	LC-MS (+)	303.2313	25.54	3.72	272.38	1.90	<0.001
	docosapentaenoic acid	LC-MS (+)	331.2627	27.25	12.43	1143.18	3.64	0.014
	14-hydroxydocosahexaenoic acid (14-HDoHE)	LC-MS (+)	357.2411	25.55	18.12	1711.84	4.18	0.021
	epoxyeicosatrienoic acids	LC-MS (−)	319.2276	21.05	14.97	1396.95	3.90	0.009
	decadienal	LC-MS (+)	153.1267	12.73	1.44	43.95	0.53	0.002
	tetradecadienal	LC-MS (+)	209.1896	12.44	1.56	55.58	0.64	0.001
	octadecadienal	LC-MS (+)	265.2508	25.92	2.02	102.42	1.02	0.027
	anandamide	LC-MS (+)	322.2742	27.94	1.76	76.22	0.82	0.003
	undecanoylcarnitine	LC-MS (+)	330.2640	8.56	0.58	−41.87	−0.78	0.002
	9-hydroxyoctadecadienoic acid (9-HODE)	LC-MS (−)	295.2279	18.88	2.90	189.54	1.53	0.013
Glycerolipids	MG(18:2(9,12))	LC-MS (+)	355.2844	25.33	3.32	232.14	1.73	0.012
Glycerophospholipids	PA(P-32:1)	LC-MS (−)	629.4537	27.98	2.10	109.64	1.07	0.050
	PC(18:1)	LC-MS (+)	536.3337	11.02	17.78	1678.49	4.15	0.005
Organic acids and derivatives	lactic acid	GC-MS	117.1	6.85	0.57	−43.33	−0.82	0.004
	2-hydroxybutyric acid	GC-MS	131.1	7.79	1.35	35.04	0.43	0.033
	pyruvic acid	GC-MS	174.1	6.70	0.67	−33.43	−0.59	0.010
	dechloroethylifosfamide	LC-MS (+)	236.9952	0.61	1.24	23.71	0.31	0.002
	proline	LC-MS (−)	114.0548	0.66	1.24	24.24	0.31	<0.001
	N-undecanoylglycine	LC-MS (+)	244.1914	29.89	1.79	79.35	0.84	0.042
	dynorphin B (6–9)	LC-MS (−)	604.3245	11.66	11.59	1059.01	3.53	0.017
	gluten exorphin A5	LC-MS (+)	601.2629	0.99	1.44	44.43	0.53	0.047
	succinylacetoacetate	LC-MS (−)	201.0372	0.68	0.51	−49.02	−0.97	0.040
Organoheterocyclic compounds	hydroxyhemin	LC-MS (+)	616.1771	11.56	0.36	−63.58	−1.46	0.002
	oxo-bilirubin	LC-MS (+)	599.2533	9.87	1.77	76.73	0.82	0.014
	biliverdin	LC-MS (+)	583.2544	13.17	2.43	142.76	1.28	0.022
Organic oxygen compounds	mannose	GC-MS	205.1	17.22	1.24	24.46	0.32	0.045
Prenol Lipids	retinal	LC-MS (+)	285.2222	25.55	9.35	834.69	3.22	0.013
Sterol Lipids	cholesterol	GC-MS	129.1	27.57	1.25	25.42	0.33	0.044
	androstanediol	LC-MS (+)	293.2458	24.19	18.10	1710.43	4.18	0.007
	pregnenolone	LC-MS (+)	317.2472	25.00	13.19	1219.35	3.72	0.014

%Δ, percentage of change; FC, fold change (case/control); pBH, Benjamini–Hochberg adjusted *p*-value; MG, monoacylglycerol; PA, phosphatidic acid; PC, phosphatidylcholines; RT, retention time; VIP, variable importance in the projection. FC was calculated as the ratio of the mean metabolite abundance in the breast cancer cohort to that of the healthy control group. %Δ was calculated as: [(mean BREAST CANCER − mean CONTROL)/mean CONTROL] × 100, with positive values indicating increased abundance and negative values indicating decreased abundance in the cancer group relative to the control group.

**Table 2:** Correlation between metabolites and levels of plasma 4-HNE-protein adducts.

Compound	Healthy Controls		Breast Cancer Patients	
	r	*p*	r	*p*
14-HDoHE	0.216	0.641	0.669 **^,^***	0.001
androstanediol	−0.381	0.352	0.616 **^,^***	0.001
caproic acid (hexanoic acid)	−0.405 *	0.027	−0.169	0.296
docosapentaenoic acid	−0.377	0.461	0.590 **^,^***	0.004
dynorphin B (6–9)	−0.057	0.839	0.531 **^,^***	0.002
eicosapentaenoic acid	−0.054	0.786	0.527 **^,^***	0.001
epoxyeicosatrienoic acids	−0.347	0.327	0.605 **^,^***	0.000
epoxyoctadecenoic acid/oxooctadecadienoic acid/hydroxyoctadecadienoic acid	−0.221	0.299	0.411 *^,^***	0.011
lactic acid (2-hydroxypropanoic acid)	−0.430 *	0.018	0.037	0.819
MG(18:2(9,12))	−0.099	0.611	0.348 *^,^***	0.028
Oxidized CDP-DG	0.416	0.068	0.423 **^,^***	0.007
PC(18:1)	−0.628	0.070	0.555 **^,^***	0.003
pregnenolone	−0.043	0.907	0.609 **^,^***	0.001
retinal	−0.107	0.683	0.520 **^,^***	0.002
tetradecenoylcarnitine	−0.513 **	0.005	0.041	0.816

r, Spearman correlation coefficient; Significance: * *p* < 0.03; ** *p* < 0.01, *** pBH < 0.05.

## Data Availability

The data presented in this study are available on request from the corresponding author due to ethical reasons.

## Supplementary Materials

The following supporting information can be downloaded at: https://www.mdpi.com/article/10.3390/antiox15020265/s1, Table S1: Baseline demographic and clinical characteristics of 41 breast cancer patients; Table S2: Significantly altered unknown metabolites detected by LC-MS, between patients with breast cancer and healthy control subjects; Figure S1: Permutation analysis plotting R2 and Q2 from 500 permutation tests in the OPLS-DA model. Plots were obtained using SIMCA-P+ software (version 15.0.2.5959, Umetrics, Umea, Sweden); Figure S2: The scatter plots of the cross-validated (CV) score vectors for OPLS-DA models. Plots were obtained using SIMCA-P+ software (version 15.0.2.5959, Umetrics, Umea, Sweden).
