# Supplementary material for: Integrative Analysis of 4-Hydroxynonenal-Modified Proteins and Plasma Metabolome in Breast Cancer Patients
Source: Antioxidants (Basel). 2026 Feb 21;15(2):265. doi: 10.3390/antiox15020265 (PMC12938520; doi:10.3390/antiox15020265)
Supplement: Supplementary file 1 [file antioxidants-15-00265-s001.zip › Supplementary figures.pdf]

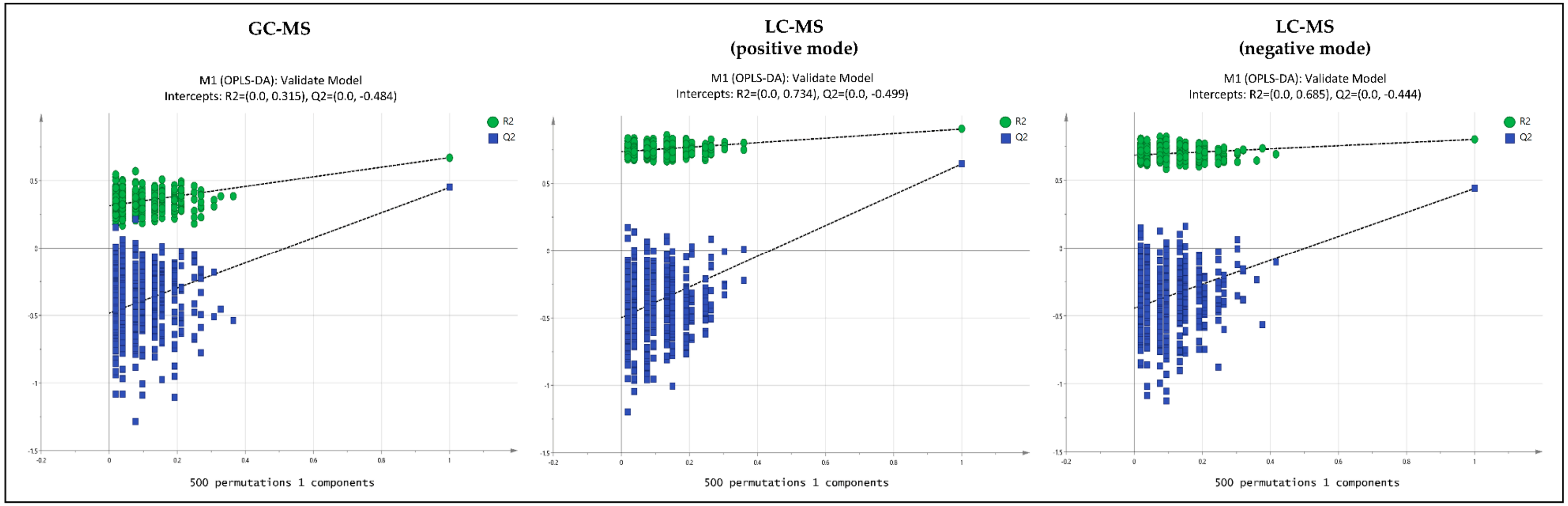

Figure S1. Permutation analysis plotting R2 and Q2 from 500 permutation tests in the OPLS-DA model. Plots were obtained using SIMCA-P+ software (version 15.0.2.5959, Umetrics, Umea, Sweden).

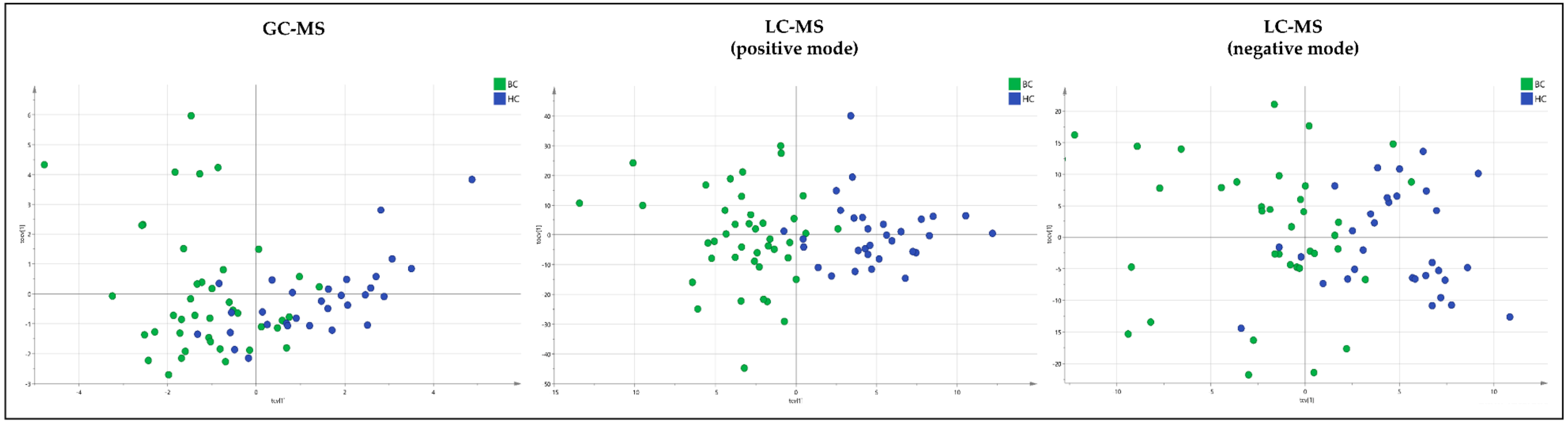

Figure S2. The scatter plots of the cross-validated (CV) score vectors for OPLS-DA models. Plots were obtained using SIMCA-P+ software (version 15.0.2.5959, Umetrics, Umea, Sweden).
