# Supplementary material for: Integrative Analysis of 4-Hydroxynonenal-Modified Proteins and Plasma Metabolome in Breast Cancer Patients
Source: Antioxidants (Basel). 2026 Feb 21;15(2):265. doi: 10.3390/antiox15020265 (PMC12938520; doi:10.3390/antiox15020265)
Supplement: Supplementary file 1 [file antioxidants-15-00265-s001.zip › Supplementary Table S1.pdf]

Supplementary Table S1. Baseline demographic and clinical characteristics of 41 breast cancer patients

**Age, years**	
Median (IQR)	57 (50-67.5)
**Menopausal status**	
Postmenopausal	N=28 (68.3%)
Premenopausal	N= 10 (24.4%)
Perimenopausal	N=2 (4.9%)
Unknown	N=1 (2.4%)
**BMI**	
Underweight (< 18.5)	N=1 (2.4%)
Normal weight (18.5 – 24.9)	N=18 (43.9%)
Overweight (25.0 – 29.9)	N=15 (36.6%)
Obesity (≥ 30)	N=5 (12.2%)
Unknown	N=2 (4.9%)
**Smoking**	
Yes	N=5 (12.2%)
No	N=32 (78.0%)
Unknown	N=4 (9.7%)
**Alcohol**	
Yes	N=1 (2.4%)
No	N=34 (82.9%)
Unknown	N=6 (14.6%)
**Corticosteroid therapy**	
Yes *	N=1 (2.4%)
No	N= 40 (97.6%)
**Diabetes mellitus**	
Yes	N=2 (4.9%)
No	N=35 (85.4%)
Unknown	N=4 (9.7%)
**Invasive breast cancer**	39 (95.1%)
**DCIS**	2 (4.9%)
**Subtype of invasive breast cancer**	
HR-positive, HER2-negative	N=19 (48.7%)
HER2-positive (HR-positive)	N=11 (28.2%)
HER2-positive (HR-negative)	N=2 (5.1%)
Triple negative breast cancer	N=7 (17.9%)
**Stage (TNM)**	
0	N=2 (4.9%)
I	N=11 (26.8%)
II	N=21 (51.2%)
III	N=2 (4.9%)
IV	N=5 (12.2%)
**Grade**	
I	N=6 (14.6%)
II	N=18 (43.9%)
III	N=12 (29.3%)
N/A	N=5 (12.2%)

Abbreviation: BMI, Body mass index; HR, hormone receptor; HER2, human epidermal growth factor 2; IQR, interquartile range; N, number

*inhalation corticosteroid therapy
