# Supplementary material for: Integrative Analysis of 4-Hydroxynonenal-Modified Proteins and Plasma Metabolome in Breast Cancer Patients
Source: Antioxidants (Basel). 2026 Feb 21;15(2):265. doi: 10.3390/antiox15020265 (PMC12938520; doi:10.3390/antiox15020265)
Supplement: Supplementary file 1 [file antioxidants-15-00265-s001.zip › Supplementary Table S2.pdf]

**Supplementary Table S2.** Significantly altered unknown metabolites detected by LC-MS, between patients with breast cancer and healthy control subjects.

| Compound | ESI mode | m/z | RT | %Δ | FC | $log_2FC$ | pBH | VIP |
|---|---|---|---|---|---|---|---|---|
| Unknown | + | 617.2598 | 9.87 | 79.64 | 1.80 | 0.85 | < 0.05 | 1.22 |
| Unknown | + | 560.3344 | 11.59 | 2339.87 | 24.40 | 4.61 | < 0.001 | 5.42 |
| Unknown | + | 339.3411 | 30.75 | 165.71 | 2.66 | 1.41 | < 0.0001 | 3.10 |
| Unknown | + | 316.3208 | 14.96 | | | | < 0.0001 | 8.42 |
| Unknown | + | 316.3208 | 14.33 | | | | < 0.0001 | 7.56 |
| Unknown | + | 296.2954 | 31.21 | -100.00 | 0.00 | | < 0.0001 | 5.13 |
| Unknown | + | 1098.5687 | 0.94 | 458.01 | 5.58 | 2.48 | < 0.0001 | 4.14 |
| Unknown | - | 940.4520 | 0.97 | 212.05 | 3.12 | 1.64 | < 0.001 | 6.90 |
| Unknown | - | 637.2264 | 9.91 | 72.67 | 1.73 | 0.79 | < 0.05 | 2.23 |
| Unknown | - | 615.2452 | 9.92 | 75.04 | 1.75 | 0.81 | < 0.05 | 1.64 |
| Unknown | - | 146.9801 | 0.68 | -41.56 | 0.58 | -0.78 | < 0.05 | 1.04 |

%Δ, percentage of change; FC, fold change (case/control); pBH, Benjamini-Hochberg adjusted p-value; MG, PA, phosphatidic acid; PC, phosphatidylcholines; RT, retention time; VIP, variable importance in the projection
